# Antioxidant activity and hepatoprotective potential of agaro-oligosaccharides in *vitro *and in *vivo*

**DOI:** 10.1186/1475-2891-5-31

**Published:** 2006-12-02

**Authors:** Haimin Chen, Xiaojun Yan, Peng Zhu, Jing Lin

**Affiliations:** 1 Key Laboratory of Marine Biotechnology, Ningbo University, Ningbo, 315211, P. R. China

## Abstract

**Background:**

Agaro-oligosaccharides derived from red seaweed polysaccharide have been reported to possess antioxidant activity. In order to assess the live protective effects of agar-oligosaccharides, we did both *in vitro *and *in vivo *studies based on own-made agaro-oligosaccharides, and the structural information of this oligosaccharide was also determined.

**Method:**

Structure of agaro-oligosaccharides prepared with acid hydrolysis on agar was confirmed by matrix-assisted ultraviolet laser desorption ionization time of flight mass spectrometry (MALDI-TOF-MS) and NMR. The antioxidant effect of agaro-oligosaccharides on intracellular reactive oxygen species (ROS) was assessed by 2', 7'-dichlorofluorescin diacetate. Carbon tetrachloride was used to induce liver injury, some index including SOD, GSH-Px, MDA, AST, ALT were examined to determine the hepatoprotective effect of agaro-oligosaccharides.

**Results:**

Agaro-oligosaccharides we got were composed of odd polymerizations with molecular weights ranged from 500 to 2500. Results from intracellular test indicated that agaro-oligosaccharides could significantly scavenge the level of oxidants in the hepatocytes, more beneficially, also associated with the improvement of cell viability *In vivo *studies of the antioxidant effects on tissue peroxidative damage induced by carbon tetrachloride in rat model indicated that agaro-oligosaccharides could elevate the activity of superoxide dismutase (SOD), glutathione peroxidase (GSH-Px) and decrease the level of malondialdehyde (MDA), glutamate oxaloacetate transaminase (AST), glutamic pyruvic transaminase (ALT) significantly. At 400 mg/kg, MDA level reduced 44 % and 21 % in liver and heart, SOD and GSH-Px increased to highest in liver and serum, while ALT level decreased 22.16 % in serum.

**Conclusion:**

Overall, the results of the present study indicate that agaro-oligosaccharides can exert their *in vitro *and *in vivo *hepatoprotective effect through scavenging oxidative damage induced by ROS.

## Background

Liver is the main organ involved in the metabolism of biological toxins and medicinal agents. Such metabolism is always associated with the disturbance of hepatocyte biochemistry and generation of ROS (reactive oxygen species) [1]. Lots of liver damages ranging from subclinical icteric hepatitis to necroinflammatory hepatitis, cirrhosis, and carcinoma have been proved to associate with the redox imbalance and OS (oxidative stress) [2]. Therefore, a potential novel approach, namely developing antioxidant drugs to treat and protect liver injury and liver disease, has been proposed [3]. This strategy is aimed to devise and incorporate antioxidants into the therapeutic for control of viral infections or protecting body from alcohol or other toxin damage. We think antioxidants are able to reduce hepatic inflammation and fibrosis, thus slowing or even preventing progression to cirrhosis. One of such candidates is agaro-oligosaccharides prepared from agar, which was chosen in the present study.

Agar was easily extracted from red algae and widely be used as food and gelling agent with historic record of more than a thousand years in China and Japan. In recent years, agaro-oligosaccharides which derived from agarose have been widely investigated in structures and bioactivities [4-8]. Many beneficial health properties of agaro-oligosaccharides are attributed to their antioxidant activities. For example, agaro-oligosaccharides have been proved to possess antioxidative activities in scavenging hydroxyl free radical, scavenging superoxide anion radical and inhibiting lipid peroxidation in various chemical assays [9-11]. Enoki *et al*. [12] also reported that the agarobiose shows the ability to suppress the expression of iNOS (inducible nitric oxide synthase), an enzyme associated with the production of NO. In our previous work, we also discussed the indirect attenuate effect of agaro-oligosaccharides towards oxidation of human liver cells induced by antimycin A [13]. These reports exhibited the potential prospects of agaro-oligosaccharides as functional ingredient to prevent the ROS related diseases. However, no researches have been done about their antioxidant effect in the *in vivo *system. Therefore, in order to evaluate the ROS scavenging activity of agaro-oligosaccharides as well as possible liver injury protection from OS with the respects of degree of polymerization, we firstly prepared agaro-oligosaccharides with different degrees of polymerizations, then use the compounds to examine the *in vitro *and *in vivo *antioxidant effects depending on hepatocyte cellular assay of H_2_O_2 _induced damage and experimental rat model of carbon tetrachloride (CCl_4_) induced toxic hepatitis.

## Methods

### Preparation of agaro-oligosaccharides

Agaro-oliogsaccharides were prepared by acid hydrolysis. In order to evaluate the difference of DP of oligosaccharides on bioactivity, hydrolysis solution was fractionated by activated carbon column. After loading the hydrolysate onto column, the column was washed with 2 liters water to remove salts and monosaccharides. Followed this step, the agaro-oligosaccharides fraction was eluted sequentially with 8 %, 15 % and 25 % hydroalcoholic solution. Each fraction from the column was concentrated under reduced pressure and lyophilized.

### Structural information of agaro-oligosaccharides

The average molecular weight of three fractions was measured as described by Somogyi et al. [14].

The nuclear magnetic resonance (NMR) spectra were acquired on an AVANCEDMX-500-NMR spectrometer. Samples were dissolved in D_2_O. ^13^C NMR spectra of 4% (w/v) solutions were recorded at 35°C under 100.69 MHz. Proton decoupled ^13^C NMR chemical shifts were measured in parts per million. For ^1^H-NMR, samples (7–10 mg) were dissolved in D_2_O (0.5 ml), and spectra were recorded at room temperature using a spectral width of 5.7 kHz, 90° pulse, an acquisition time of 4.4 s for 144 scans.

Mass spectrometry analysis was performed on a Bruker Reflex III MALDI-TOF-MS (Bruker-Daltonik, Germany) in the delayed extraction and positive mode. An accelerating voltage and a reflectron voltage were set at 20 kV of 22.8 kV, respectively, during the measurements. 2, 5-Dihydroxybenzoic acid was used as matrix (20 mg/ml; 3:2 water/MeCN) and approximately 10–100 pg of the DP-H agaro-oligosaccharide mixture was deposited as a mixture together with the matrix on a stainless steel target, and subsequently dried under reduced pressure. During the experiments, the laser power was adjusted to a level just above the threshold for formation of observable ions. The results from 20 to 100 laser shots were summed for sample.

### Measurement of intracellular ROS generation

Intracellular oxidant stress was monitored by measuring changes in fluorescence resulting from intracellular probe oxidation.

Human hepatocyte L-02 purchased from Chinese Institute of Biochemistry and Cell Biology was cultured in RPMl-1640 medium with 20 % fetal bovine serum. Viable cells (10^5^/ml) were plated into a 96-well for 1 day. On the day of the experiments, after removing the medium, the cells were washed with PBS for three times and then incubated with different doses of agaro-oligosaccharides in 5 % CO_2 _at 37°C for 2 h. After incubation, 20 μM DCFH-DA was added for another 45 min. The DCFH-DA was removed by washing the cells with PBS. 100 μM H_2_O_2 _were added into cells for 45 min and the fluorescence change was monitored by fluorescence spectorphotometer at λ_ex _= 475 nm, λ_em _= 525 nm [15].

### Cell viability and cytotoxicity assessment

The cell viability was quantified using MTT assay. Briefly, 1 × 10^4 ^cells were seeded in each well of microtiter plate and allowed to attach overnight. Cells were treated with various doses of agaro-oligosaccharides for different period according to the experiment purpose. For cytotoxicity test, the hepatocyte L-02 was treated for 48 h. But for the detection of protective effect of agaro-oligosaccharides on H_2_O_2 _damage, the L-02 was only treated for 2 h, and then 100 μM H_2_O_2 _was added for another 2 h. MTT in PBS was added to each well, followed by incubation for 4 h at 37°C. The formazan crystals were dissolved in DMSO. The optical density was determined with a microculture plate reader at 492 nm [16].

### Animals model

Mature Wistar rats weighing 150 ± 20 g were supplied by the animal center of Hangzhou, China. The animals were housed in a room with a 12 h light/dark cycle at about 22°C and fed on standard diet with ad libitum access to drinking water. All treatments were conducted between 9:00 am and 10:00 am to minimize variations. In this study, rats were randomly divided into six groups. Group 1 (control, n = 8): water for 10 days followed by administration of liquid paraffin only; group 2 (CCl_4_, n = 8): water for 10 days followed by administration of CCl_4 _on the final day; group 3 (positive control, n = 8): vitamin C (200 mg/kg) + CCl_4_; group 4 to 6 (n = 8): agaro-oligosaccharides (200, 400, 600 mg/kg, respectively) + CCl_4_. Rats were injected i.p. with vitamin C or agaro-oligosaccharides for ten consecutive days. On the final day, all animal except control group were administered with 20 % CCl_4 _in liquid paraffin at a dose 5 ml/kg to induce hepatotoxicity. Previous studies demonstrated that the OS indexes could reach a maximum at 48 h after CCl_4 _i.p. administration [17], therefore, in this work rats were sacrificed by collecting the blood from the carotid artery after 48 h of administration. Two organs (liver and heart) were excised immediately.

### Biochemical assays

Serum was separated by centrifugation at 1000 × g at 4°C for 10 min. 10 % organ homogenates including liver and heart were prepared in ice-cold isotonic physiological saline. The GSH-Px, MDA, SOD, AST and ALT levels of tissue and serum were measured by spectrophotometric methods as described in the assay kits.

### Statistical analysis

All data are expressed as mean ± SD. In cell based assay, the control and agaro-oligosaccharides treated cells were compared by student *t*-test. In animal assay, the statistical tests were one-way ANOVA followed by post-hoc Newman-Keuls multiple comparisons test. A probability level of 0.05 was considered statistically significant.

## Results

### Preparation and structure analysis of agaro-oligosaccharides

Activated charcoal column has been performed as saccharide isolation tool for decades. Depending on this technology, we successfully achieved to isolate three fractions of agaro-oligosaccharides with average molecular weight of 619, 1126 and 1631, respectively, eluted by 8 %, 15 % and 25 % aqueous alcoholic solution. We use these three fractions for the following experiments, designated as DP-L, DP-M and DP-H, according to their differences in molecular weight.

**Figure 1:**
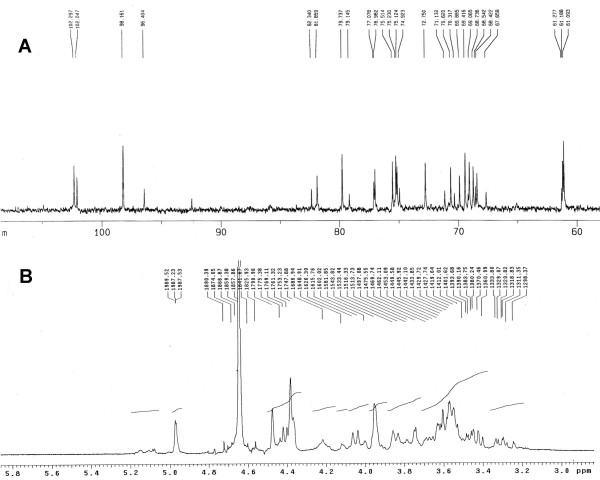
^1^H-NMR and ^13^C-NMR spectra of solid acid hydrolysate.

Since agar is a linear copolymer of galactose (G), alternated with 3, 6-anhydrogalactose (A), the structural difference of agaro-oligosaccharides are mainly related with the degree of polymerization. In this report, the ^1^H-NMR and ^13^C-NMR spectra of agaro-oligosaccharides was studied using acid hydrolyzed fragments, and typical deshielded ^1^H-NMR and ^13^C-NMR signals corresponding to the anomeric hydrogens and carbons were obtained and presented in Fig. 1. Assignments were based on the close similarity with literature values, and the interpretation of these signals was indicated in Table 1. The spectra give out twelve distinctive major anomeric carbon signals which were expected for the major disaccharide repeat unit. The presence of these signals demonstrates the presence of floridean starch in this fraction, because all of the signals illustrated the galactose ring structures present in seaweed galactans [18,19]. The ^13^C NMR spectrum of oligosaccharide are very consistent with those previously published for neoagarose series, with chemical shifts of carbons of unit G'-1α and G'-1β appeared at identically 92.4 and 96.4 ppm, having intensities in the ratio of 1 : 2. While, from the result, we didn't observe any signal for 3, 6-anhydrogalactose at the non reducing ending (a peak at 91.4 ppm) [20-22]. These results reflect the presence of galactose units at the reducing ends of the reaction products, but no 3, 6-anhydrogalactose at non-reducing end.

**Table 1:** Chemical shift assignments for ^1^H-NMR and ^13^C-NMR spectra of agaro-oligosaccharides

Unit		Chemical shifts (ppm)					
		
		C-1	C-2	C-3	C-4	C-5	C-6
Carbon	G ^a^	102.3	70.6	82.3	68.7	75.2	61.2
	A ^b^	98.1	69.9	79.7	77.1	75.5	69.4
Proton	G	4.39	3.79	3.6	4.12	3.55	3.63 ^c^/3.67 ^d^
	A	4.97	3.96	4.37	4.48	4.4	3.84 ^e^/4.06 ^f^

a galactose.

b anhydrogalactose.

c A-6 exo proton

d A-6'endo proton

e G-6 proton

f G-6' proton

**Figure 2:**
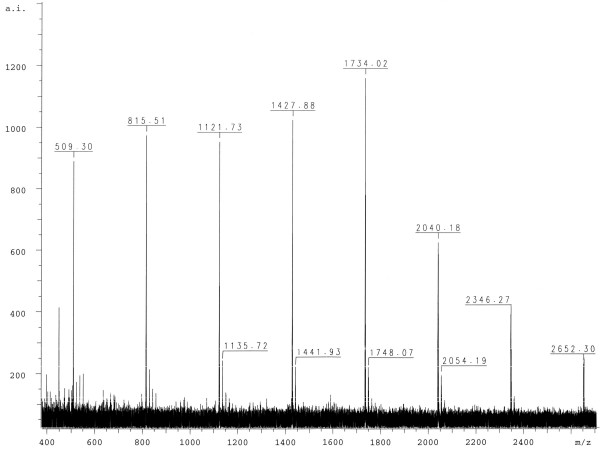
MALDI-TOF mass spectrum of agar hydrolysate. (m/z 400–2700).

We applied MALDI-TOF-MS in order to know the structural information of composition and DP in the agaro-oligosaccharide fractions which were obtained from hydrolysis. The results shown in Fig. 2 indicated that a large number of well-regulated peaks are present, and these agaro-oligosaccharide ions could be identified as series of sodium molecular ions with relatively high intensities corresponded to 509, 815, 1121, and so on. It can be seen, by comparing these ions, that the molecular mass difference between every two adjacent ion is the same as 306 Da. This molecular mass difference of 306 Da is the exact molecular mass of agaro-biose (GA), the basic structural unit, which is 324 Da, minus 18, which is the number of H_2_O's molecular weight, therefore, it is clearly observed that the agaro-oligosaccharides had very regular molecular structures with gradient increase of its chain length with the polymerization unit of agarobiose. Further calculation for m/z 509, the lowest high intensity ion observed in the mass spectrum, found that m/z 509 corresponds to the sodium adduct of agarotriose (GAG) [M_tri_+Na]^+^. Based on this information, the ion at m/z 815, 1121, 1427.... corresponds to agaro-oligosaccharides for n = 5, 7, 9..., respectively with galactose at both reducing end and non-reducing end. For agaro-oligosaccharides, two forms of saccharides exist depending on the end sugar moiety, namely, neoagaro-series with 3, 6-anhydro-galactose at the non-reducing end and agaro-series with galactose at the non-reducing end. The results obtained here indicated that our sample obtained belong to agaro-series with odd numbers of sugar unit.

### The antioxidant action of agaro-oligosaccharides in cell based assay

We firstly investigated the antioxidant activities of agaro-oligosaccharides in the cellular system. DCFH-DA, which can be conversed from non-fluorescence into fluorescence through oxidation, was used as fluorescent probe to monitor the changes of oxidative stress in hepatocyte L-02 induced by addition of H_2_O_2_. In our experiment, all the measurements were carried out at the steady stage (incubation time, 60 min) in order to minimize variations, because it has been reported that treatment of H_2_O_2 _will lead to the abruption of ROS in few minutes, and then decrease to a steady stage [23].

**Figure 3:**
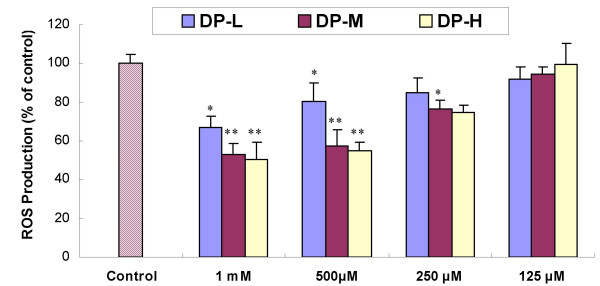
**Effect of agaro-oligosaccharides on DCF fluorescence in hepatocytes**. Values expresses as mean ± SD. n = 6. * P < 0.05, ** P < 0.01, vs control cells without sample.

**Figure 4:**
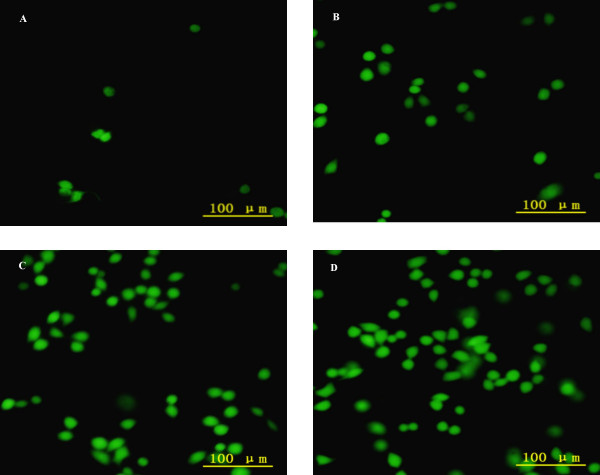
**Inhibition of intracellular oxidant by agaro-oligosaccharides**. (A) Control without H_2_O_2_, (B) 1 mg/ml 25% ethanol eluted fraction, (C) 125 μg/ml 25% ethanol eluted fraction, (D) Positive control.

Fig. 3 showed that addition of agaro-oligosaccharides caused concentration-dependent attenuation of DCF fluorescence. Three groups of agaro-oligosaccharides showed almost no inhibitory activity at the 125 μg/ml. When the concentration increased, DP-H expressed highest activity, followed by DP-M, much weaker for DP-L group, which indicating that the antioxidant bioactivity *in vitro *improves with the higher degree of polymerization of agaro-oligosaccharide. Fig. 4 is a typical fluorescent microscopic picture of the DCF fluorescence in hepatocyte L-02 treated with DP-H. It is obvious that H_2_O_2 _lead to the production of ROS, which transformed the DCFH into DCF (Fig. 4D), showing more fluorescent cells than untreated cells (Fig. 4A). DP-H additions decreased the free radical formation. Fig. 4B clearly illustrated that DP-H at concentration of 1 mg/ml could inhibit the oxidation of DCFH significantly. While with the concentration decreased to 125 μg/ml (Fig. 4C), the number of fluorescent cells was also increased, and which means that the antioxidant activity of agaro-oligosaccharides acts in a concentration-dependent manner.

### Protective effect of agaro-oligosaccharides on oxidative stress injury

**Figure 5:**
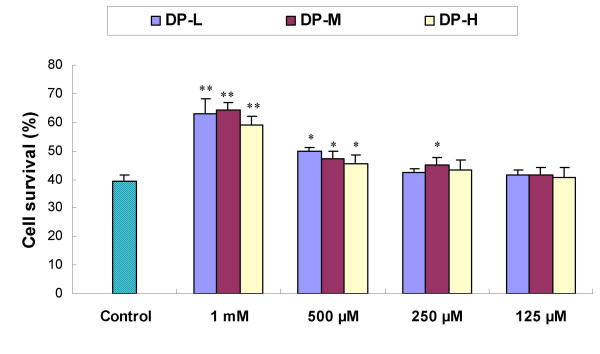
**Effect of agaro-oligosaccharides on cell survival during H_2_O_2 _exposure**. Values expresses as mean ± SD. n = 6, * P < 0.05, ** P < 0.01, vs control.

Oxidative stress is an important factor to induce the cell death. Cell viability assay showed that the presence of H_2_O_2 _(100 μM) resulted in cell death ratio increasing to 60 % after 2 h of treatment (Fig. 5). Compared to H_2_O_2 _alone, cell death was reduced obviously when exposed to each agaro-oligosaccharide group at the higher concentrations (from 500 μg/ml to 1 mg/ml). The cell viability significantly increased to 64.26 % for DP-M treated cells at the concentration of 1 mg/ml (Fig. 5). At low concentrations (125 μg/ml to 500 μg/ml), there was almost no variation observed between the agaro-oligosaccharide treated cells and the control, except DP-M treated group showing some weak cell protective effect. These results demonstrated that the antioxidant activities of agaro-oligosaccharides were positively correlated with the improvement of the cell viability.

**Figure 6:**
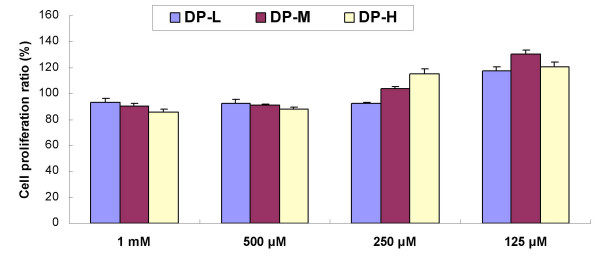
**Effects of different concentrations of agaro-oligosaccharides on cell proliferation after exposure of cells for 48 h**. Values expresses as mean ± SD. n = 3

In order to test whether agaro-oligosaccharides affected the growth of human hepatocyte L-02 without H_2_O_2 _treatment, cell proliferation was assessed by direct MTT assay. The cells were incubated with various amounts of agaro-oligosaccharides for 48 h, and the change in cell number was determined by analyzing the values of cells treated with agaro-oligosaccharide versus that of control (Fig. 6). From result, we found that compared with control group, the agaro-oligosaccharides exhibited very slight effects on the cell growth. After 48 h of treatment, the growth is slightly inhibited as of 14.18 % for DP-H at 1 mM, while for DP-M and DP-H, the corresponding cell proliferation ratio was >100 % with concentration ≤ 250 μM, which means that, at proper concentration, the agaro-oligosaccharides can promote the proliferation of L-02 cells. Therefore, the cell survival effect of agaro-oligosaccharide alone in the antioxidation cellular assay can be considered almost naught because the cells were only treated for 2 h, so we concluded that agaro-oligosaccharides can effectively protect the cells from oxidation induced death through scavenging intracellular oxidative damage induced by ROS.

### Effect of agaro-oligosaccharides on an acute CCl_4 _oxidative damage

We further studied the *in vivo *antioxidant effects of agaro-oligosaccharides. It was not uncommon that compounds possessing *in vitro *activity, however, fail to maintain the activity when administrated into body. We established an oxidative animal model by CCl_4 _injection. Considering the proliferation and antioxidant effects of agaro-oligosaccharides on hepatocyte, we used the mixture of DP-M and DP-H as our sample for animal test. The effects of agaro-oligosaccharides on oxidative stress in rats were estimated by determining the activities of MDA, SOD, GSH-Px, ALT and AST in serum and tissues.

MDA level is a main marker of endogenous lipid peroxidation [24]. In CCl_4 _treated group, the MDA level increased significantly in liver (F = 2.087, P < 0.05), but little difference was observed in serum, which confirmed that the toxicity of CCl_4 _is focused in the liver. By contrast, MDA level in the agaro-oligosaccharides treated groups decreased significantly compared with CCl_4 _treated group. At 400 mg/kg, the MDA level reduced at least 44 % and 21 % in liver (F = 4.274, P < 0.05) and heart, respectively, versus the CCl_4 _treated group. Actually, the MDA level of agaro-oligosaccharides treated groups showed almost the same as the blank control group (Table 2). It provided the information that exhibiting a very successful block of lipid oxidation.

**Table 2:** Effect of agaro-oligosaccharides on MDA activity in different organs of CCl_4 _induced rats ^a^

Groups	Liver (nmol/mg prot)	Heart (nmol/mg prot)	Serum (nmol/ml)
Normal control	2.75 ± 0.51	0.56 ± 0.08	4.20 ± 0.22
CCl_4 _control	4.62 ± 0.77^#^	0.68 ± 0.05	4.44 ± 0.64
Vitamin C	2.59 ± 0.02*	0.67 ± 0.11	3.53 ± 0.74
G4 (200 mg/kg)	3.45 ± 0.77	0.54 ± 0.11	3.33 ± 0.11
G5 (400 mg/kg)	2.71 ± 0.18*	0.53 ± 0.14	3.36 ± 0.63
G6 (600 mg/kg)	2.99 ± 0.47	0.45 ± 0.02	2.82 ± 0.66

^a ^n = 8. Each value represents the mean ± SD.

Significant values: *P < 0.05 (*vs *CCl_4 _group); ^#^P < 0.05 (*vs *Normal group).

G4, G5 and G6: group 4, 5, 6 which administrated with sample of 200 mg/kg, 400 mg/kg and 600 mg/kg, respectively.

SOD and GSH-Px are intracellular antioxidant enzymes that protect against oxidative process [25]. As show in Table 3 and 4, a single high dose injection of CCl_4 _induced severe oxidative damage and the SOD and GSH-Px level decreased markedly. While various concentrations of agaro-oligosaccharides could effectively normalize the enzyme activities and the two indexes were even higher than Vitamin C group. In liver and serum, the SOD level reached to highest at 400 mg/kg (F = 3.878, P < 0.05; F = 9.363, P < 0.05). Similar results were obtained in case of the GSH-Px activities.

**Table 3:** Effect of agaro-oligosaccharides on SOD activity in different organs of CCl_4 _induced rats ^a^

Groups	Liver (U/mg prot)	Heart (U/mgprot)	Serum (U/ml)
Normal control	34.18 ± 2.45	46.80 ± 2.84	313.77 ± 24.01
CCl_4 _control	26.97 ± 6.69^#^	27.71 ± 2.26^#^	306.89 ± 19.29
Vitamin C	33.11 ± 2.79*	32.75 ± 1.73	318.35 ± 16.39
G4 (200 mg/kg)	33.45 ± 2.87*	34.37 ± 1.34	361.08 ± 9.37*^#^
G5 (400 mg/kg)	38.64 ± 8.44*	37.33 ± 2.45	365.53 ± 21.13*^#^
G6 (600 mg/kg)	35.42 ± 2.86*	40.49 ± 2.21*	364.92 ± 14.21*^#^

^a ^n = 8. Each value represents the mean ± SD.

Significant values: *P < 0.05 (*vs *CCl_4 _group); ^#^P < 0.05 (*vs *Normal group).

**Table 4:** Effect of agaro-oligosaccharides on GSH-Px activity in different organs of CCl_4 _induced rats ^a^

Groups	Liver (NU/mgprot)	Heart (NU/mgprot)	Serum (*× *10^3 ^NU)
Normal control	159.17 ± 6.97	200.20 ± 15.46	12.50 ± 1.44
CCl_4 _control	91.60 ± 3.97^#^	191.81 ± 36.90	10.38 ± 1.48^#^
Vitamin C	119.41 ± 9.86	204.86 ± 17.11	11.02 ± 0.66
G4 (200 mg/kg)	120.50 ± 17.05	203.63 ± 25.01	12.18 ± 1.95
G5 (400 mg/kg)	127.19 ± 12.17*	217.40 ± 10.82	13.13 ± 1.21
G6 (600 mg/kg)	118.92 ± 17.56	248.47 ± 39.28	12.26 ± 1.30

^a ^n = 8. Each value represents the mean ± SD.

Significant values: *P < 0.05 (*vs *CCl_4 _group); ^#^P < 0.05 (*vs *Normal group)

**Figure 7:**
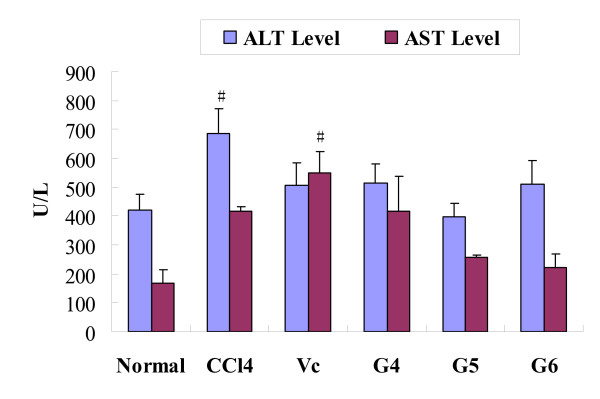
**Effect of agaro-oligosaccharides on AST and ALT activity in serum**. Values expresses as mean ± SD. n = 8, ^#^P < 0.05, vs Normal group. G4, G5 and G6: group 4, 5, 6 which administrated with sample of 200 mg/kg, 400 mg/kg and 600 mg/kg, respectively

Serum levels of transaminases (ALT, AST) were used as indicators to evaluate the attribution of agaro-oligosaccharides to the structure damage of the liver [26,27]. In this experiment, the enzyme assays of serum transaminases showed that a toxic dose of CCl_4 _significantly raised the levels of ALT and AST to 687 (F = 3.761, P < 0.05) and 415 U/l (F = 4.204, P < 0.05). Agaro-oligosaccharides could inhibit the enzyme activities effectively. The ALT level reached to minimum when the sample concentration was 400 mg/kg (22.16 % less than the control group). For AST, the agaro-oligosaccharides reduce it in a dose dependent manner. At the highest concentration (600 mg/kg), AST level decreased to 222 U/l. However, it is strange to find that Vitamin C didn't reduce AST but raised it to 32 % versus the control without CCl_4 _treatment (Fig. 7).

## Discussion

Among therapeutics for liver diseases, protective drugs have been attracted more and more attentions, such as antioxidant prevention approaches. In this paper, we focused on the *in vitro *and *in vivo *antixoidative activities of agaro-oligosaccharides with the model related with liver disease.

Agaro-oligosaccharides are linear oligomers cleaved from agar which is built of 1, 4-linked 3, 6-anhydro-α-L-galactose alternating with 1, 3-linked β-D-galactopyranose. When agar is attacked by degradation reagents, such as hydrolysis enzyme, acid or alkali, numerous possibilities for combination, viz., the repetition of AG, GA, AGA, or GAG, etc will exist. In this research, depending on NMR and MALDI-TOF-MS analysis, we detected the precise structural features of our hydrolysate. NMR results give us information that our product is agarose structure, furthermore, there was no signal of A at reducing end. In the spectrum of MALDI-TOF-MS, the first high intensity peak observed at m/z 509 was assigned to (M_tri_+Na)^+ ^containing two galactopyranose (Gal*p*) residues and one 3,6-anhydrogalactopyranose (AnGal*p*) residues, followed by a series of agaro-oligosaccharides: agaropentaose, agaroheptanose, agarononaose, and so forth. In our case, the agaro-oligosaccharides with odd polymerization degree were dominant.

For the *in vitro *antioxidant studies, we noticed that agaro-oligosaccharides expressed different antioxidant abilities with different ranges of DPs. In them, the fraction of DP-H with average MW of 1631 showed highest free radical scavenging activity which agrees well with the result obtained by Zhao et al. [10]. However, Enoki et al. [12] found, in a different assay system, that agarobiose possessed the highest ability to inhibit the expression of iNOS. Therefore, comparison of structure-bioactivity *in vitro *for different studies should be careful bearing different assays in mind.

It is quite significant that the *in vivo *animal experiment for agaro-oligosaccharides is quite consistent with the *in vitro *assays. Besides successful protection of liver damage by efficiently inhibiting MDA formation and decreasing AST and ALT, agaro-oligosaccharides enhance the activities of antioxidant enzyme system of the host, including SOD, GSH-Px. We also notice that vitamin C only slightly reduced AST and ALT level in rats in our experiment, although it prevented MDA formation effectively (Fig. 7). The result indicates that agaro-oligosaccharides have better impact to improve the hepatoprotective ability. Since antioxidant enzymes such as SOD and GSH-Px are considered to be a primary defense system for oxidative damage prevention, agaro-oligosaccharides exert antioxidant not only through its own radical scavenging activity, but also, by boost the host antioxidant enzyme system. On the other hand, we found that when the sample concentration increased from 400 mg/kg to 600 mg/kg, several indexes showed a different change. At concentration of 600 mg/kg, the MDA level increased slightly and SOD, GSH-Px and AST activities reduced a little. This result implied that excessive administration of agaro-oligosaccharides will decrease their antioxidant ability with unknown reasons.

In conclusion, by carefully examining the antioxidant protective effects of agaro-oligosaccharides both *in vitro *and in *vivo*, the agaro-oligosaccharides prepared via solid acid hydrolysis showed consistent and concentration-dependent antioxidation activities, as well as significant protection against liver injury.

## Conclusion

These results support a beneficial relationship between antioxidant activity and hepatoprotective effect of agaro-oligosaccharides which belong to agaro-series with odd numbers of sugar unit as their dominant composition.

## Abbreviations

**A**: 3, 6-anhydrogalactose

**DCFH-DA**: 2', 7'-dichlorodihydrofluorescein diacetate

**DP**: degree of polymerization

**DP-H**: Degree of Polymerization-High, representing the experiment group of the agaro-oligosaccharides with average molecular weight of 1631, eluted by 25 % ethanol from the charcoal column

**DP-L**: Degree of Polymerization-Low, representing the experiment group of agaro-oligosaccharides with average molecular weight of 619, eluted by 8 % ethanol from the charcoal column;

**DP-M**: Degree of Polymerization-Middle, representing the experiment group of agaro-oligosaccharides with average molecular weight of 1126, eluted by 15 % ethanol from the charcoal column;

**G**: galactose;

**MDA**: malondialdehyde;

**MTT**: 3-(4, 5-dimethyl-2-thiazolyl)-2, 5-diphenyl-2H-tetrazolium bromide

**MW**: molecular weight;

**OS**: oxidative stress;

**ROS**: reactive oxygen species

## Authors' contributions

HMC have been involved in drafting the manuscript.

XJY have made substantial contributions to conception and design.

ZP carried out the animal experiment.

LJ participated in the cell biology research.
